# Comparative Studies on Synthesis Methods of BiVO_4_ for Photoelectrochemical Applications

**DOI:** 10.3390/molecules30183818

**Published:** 2025-09-19

**Authors:** Dominik Caus, Katarzyna Berent, Krzysztof Mech, Andrii Naumov, Marianna Marciszko-Wiąckowska, Agnieszka Podborska

**Affiliations:** 1 Faculty of Materials Science and Ceramics, AGH University of Krakow, al. Mickiewicza 30, 30-059 Krakow, Poland; caus@student.agh.edu.pl; 2 Academic Centre for Materials and Nanotechnology, AGH University of Krakow, al. Mickiewicza 30, 30-059 Krakow, Poland; kberent@agh.edu.pl (K.B.); kmech@agh.edu.pl (K.M.); naumov@agh.edu.pl (A.N.); marciszk@agh.edu.pl (M.M.-W.)

**Keywords:** BiVO_4_, photoelectrochemistry, photocatalysis, PEPS effect

## Abstract

In this work, we report optical and photoelectrochemical properties of BiVO_4_ synthesized by microwave, sonochemical, sol–gel, and direct deposition on conductive substrate methods. Structural and morphological characterization using XRD, SEM, and AFM confirmed the presence of both monoclinic and tetragonal phases, with variations in particle size and surface roughness. UV-Vis spectroscopy revealed band gaps in the range of 2.38–2.51 eV. Photoelectrochemical performance was evaluated through measurements of photocurrents under varying illumination wavelengths and applied potentials. BiVO_4_ as a thin film exhibited the highest photocurrent intensity due to its superior semiconductor–substrate contact. In contrast, BiVO_4_ samples obtained as a powder showed significantly lower photocurrents but demonstrated the photocurrent switching effects, attributed to the presence of surface trap states and oxygen vacancies. The obtained results highlight the importance of synthesis strategy in tailoring BiVO_4_ properties for use as a photoelectrochemical cell and suggest potential applications in molecular electronics, such as logic gates and memory devices.

## 1. Introduction

The increasing global demand for energy has driven extensive research into sustainable and renewable energy sources. Among these, solar energy is one of the most promising due to its abundance and low environmental impact. Solar radiation can be converted directly into electricity through the photovoltaic effect, which occurs in semiconductor materials. As a result, scientists around the world are intensively investigating and engineering advanced semiconductors—such as perovskites [1], silicon [2], and organic photovoltaic compounds [3]—to maximize energy conversion efficiency, stability, and scalability for practical applications [4].

A significant class of semiconductors used in photovoltaic applications includes metal oxides (TiO_2_, ZnO, SnO_2_, and NiO_x_) [5], as well as metal sulfides like CdS and ZnS [6]. For many years, titanium dioxide (TiO_2_) was considered one of the most promising semiconductor materials for photovoltaic applications. However, its wide band gap of approximately 3.2 eV limits its light absorption to the ultraviolet region of the solar spectrum, significantly reducing its overall efficiency under sunlight [7,8]. Cadmium sulfide (CdS) was initially regarded as a potential breakthrough semiconductor due to its narrower band gap of approximately 2.4 eV, enabling the absorption of a broader portion of the solar spectrum compared to TiO_2_. However, its practical application is limited by its high susceptibility to photocorrosion under illumination and the environmental and health concerns associated with its cadmium content.

Bismuth vanadate (BiVO_4_) is currently regarded as one of the most promising semiconductor materials, owing to its high photoactivity and wide range of applications, including photocatalytic water splitting [9,10], CO_2_ conversion [11], and pollutant degradation, as well as photoelectrochemical systems such as solar cells and sensors [12,13,14], batteries [15], and synaptic devices and memristors [16,17].

**Figure 1:**
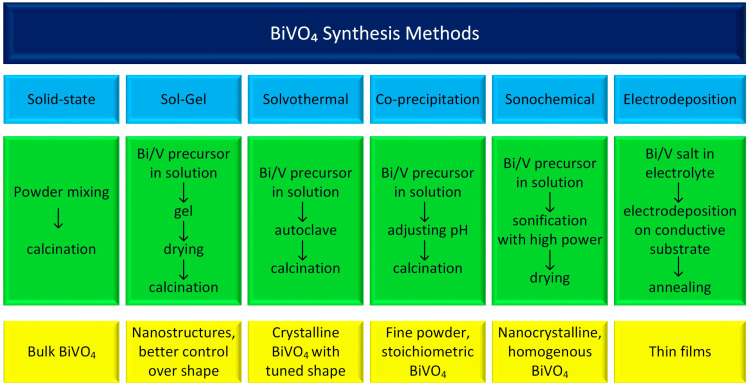
This diagram presents the most common BiVO_4_ synthesis methods.

The photochemical performance of BiVO_4_ is strongly influenced by the synthesis method, which dictates its morphology, crystallinity, phase composition (monoclinic vs. tetragonal), and photocatalytic efficiency [18,19,20]. The most common methods for synthesizing BiVO_4_ are summarized in Figure 1. While numerous other approaches exist and each method can be further modified, these variations fall outside the scope of this work. Therefore, we will focus only on the most important aspects and briefly describe the most important synthesis conditions and their advantages and disadvantages.

Solid-state synthesis typically involves mixing bismuth oxide (Bi_2_O_3_) and vanadium pentoxide (V_2_O_5_) as precursor materials, often aided by ball milling to achieve uniform blending. The resulting powder mixture is then subjected to high-temperature calcination, with typical sintering temperatures ranging from 450 °C to 800 °C. The duration of this heat treatment significantly affects the crystal structure and phase composition of the resulting BiVO_4_ powders. This method’s primary advantages are its simplicity and relatively low production cost. However, it offers limited control over particle size, phase purity, and surface area [21].

The sol–gel method is another versatile and effective technique for synthesizing BiVO_4_ with various morphologies and enhanced photocatalytic properties. Different variations of the sol–gel method, including complexing, photo-assisted, and non-hydrolytic approaches, offer unique advantages in terms of particle size, phase purity, and photocatalytic efficiency. Bismuth nitrate and ammonium vanadate are commonly used as starting materials. The materials are mixed in ethanol in the presence of a chelating agent, typically citric acid. In the first step, the materials are heated to 70 °C for 1–3 h to obtain a gel and then calcined at 400–600 °C for 2–3 h [22,23,24].

The solvothermal, co-precipitation, and sonochemical methods share similarities in substrate preparation, as all of them involve a liquid medium—typically water in the case of BiVO_4_ synthesis. However, they differ primarily in their heating techniques. The co-precipitation method is widely used due to its simplicity, low cost, and scalability. Despite these advantages, it offers limited control over particle size and chemical homogeneity [19,25]. In the solvothermal method, elevated temperature and pressure conditions promote the formation of crystalline BiVO_4_. When microwave heating is applied, the process becomes faster, more energy-efficient, and allows for uniform heating and precise temperature control. This approach also enables the use of various organic additives to influence particle size and morphology. In sonochemical synthesis, ultrasound irradiation induces acoustic cavitation in the liquid medium, generating localized high temperatures and pressures. These extreme conditions accelerate the crystallization of BiVO_4_, even at relatively low bulk temperatures [26,27].

Electrodeposition is considered an important method used to fabricate thin BiVO_4_ films for electrochemical applications. In this approach, a conductive substrate—typically indium tin oxide (ITO) or fluorine-doped tin oxide (FTO)—is immersed in a specially prepared electrolyte bath at room temperature, where deposition is carried out under a controlled potential or current. The process for BiVO_4_ generally involves multiple steps. First, bismuth is electrodeposited from an acidic aqueous solution containing Bi^3+^ ions. Subsequently, a vanadium precursor is spray-coated onto the substrate while it is heated, promoting the formation of the final BiVO_4_ phase upon further thermal treatment. This method allows for the production of highly homogeneous and stable thin films [11].

From a photoelectrochemical perspective, the crystal structure is crucial, as it determines the electronic structure of the semiconductor. There are four polymorphic forms of bismuth vanadate: the monoclinic phase, the scheelite-type tetragonal phase, the zircon-type tetragonal phase, and the orthorhombic phase [28]. According to the literature, the monoclinic phase is the most suitable for photocatalytic applications due to its high photocatalytic activity and narrow indirect band gap of approximately 2.4 eV. It is also the most thermodynamically stable phase [29,30,31]. At low synthesis temperatures, the zircon-type tetragonal phase is typically formed, characterized by a broad band gap of approximately 2.9 eV [32]. The monoclinic phase can be obtained either by calcination at temperatures above 400 °C [33] or by hydrothermal synthesis at temperatures around 180 °C [34]. Synthesis conducted at pH values lower than 3.8 may lead to the formation of the scheelite-type tetragonal phase [35], which exhibits a band gap of approximately 2.4 eV [36]. Furthermore, performing hydrothermal synthesis followed by calcination can result in the formation of the orthorhombic phase of BiVO_4_, which has a band gap of approximately 2.8 eV [37].

This study describes four different methods for the synthesis of bismuth vanadate and discusses in detail the influence of the synthesis method on the structure, morphology, and optical and photoelectrochemical properties of BiVO_4_ in the context of its various applications. Similar synthesis methods have been previously described in the literature, but until now, no one has compared the properties of the resulting materials measured under the same conditions.

## 2. Results and Discussion

### 2.1. Synthesis, Structure, and Morphology of Different BiVO_4_ Samples

In this study, we selected four different synthesis methods to determine the most optimal form of BiVO_4_ for efficient photocurrent generation: hydrothermal (m-BiVO_4_) [25], sol–gel (g-BiVO_4_) [23,24], sonochemical (s-BiVO_4_) [26,27], and thin-layer deposition (l-BiVO_4_) [38,39]. Initially, we used inorganic precursors to synthesize BiVO_4_: bismuth nitrate and sodium or ammonium orthovanadate. The substrates were dissolved in water with nitric acid to prevent bismuth oxide precipitation in the case of BiNO_3_ and in water with NaOH to prevent vanadium(V) oxide precipitation in the case of vanadate. After mixing the two solutions, the pH was adjusted to 7, and the mixture was placed in a Teflon vessel and placed in a microwave reactor for 2 h. The bismuth vanadate obtained using this method was designated as m-BiVO_4_. To test the effect of the heating method on the obtained material, an identically prepared solution was placed in the vessel and exposed to a high-power sonotrode for 90 min. During this process, the reaction mixture was continuously stirred, and the locally occurring high temperature ensured a uniform crystal pattern, thus guaranteeing that the obtained s-BiVO_4_ is homogeneous. In the next attempt, we used the sol–gel technique, adding citric acid as a chelating agent. The mixture was heated for 5 h at 90 °C, and then calcinated at 250 °C, resulting in the formation of g-BiVO_4_. All these syntheses were carried out in aqueous solutions and resulted in the precipitation of a yellow powder. In the fourth attempt, a DSMO-DMF mixture was used as the solvent and bismuth nitrate and vanadyl acetylacetonate as substrates. After mixing, a stable green solution was obtained, which was then spin-coated onto glass coated with an ITO layer and preheated until a yellow precipitate was formed. The layers (l-BiVO_4_) were then heated at 450 °C, and after cooling, the electrodes were ready for use.

**Figure 2:**
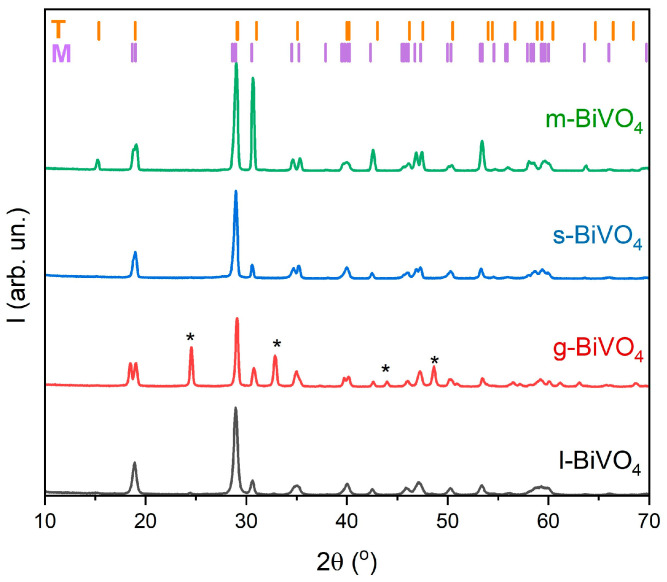
XRD analysis of BiVO_4_ samples prepared using various synthesis methods (l-, g-, s-, and m-BiVO_4_).

The phase composition of all BiVO_4_ samples was investigated using X-ray diffraction (XRD). Bismuth vanadate can crystallize into two polymorphic forms, i.e., monoclinic and tetragonal, and it is often present as a mixture of these two phases. In the case of our samples, all materials are composed of the monoclinic phase (space group: C 1 2/c 1, ICSD: 98-018-1561) [29] and the tetragonal phase (space group: I 41/a, ICSD: 98-006-2706) [40]. Notably, for the g-BiVO_4_ sample, additional diffraction peaks corresponding to monoclinic V_3_O_7_ [41] (space group: C 1 2/c 1, ICSD: 98-000-2338) were observed. These peaks are marked with an asterisk (*) in Figure 2.

**Figure 3:**
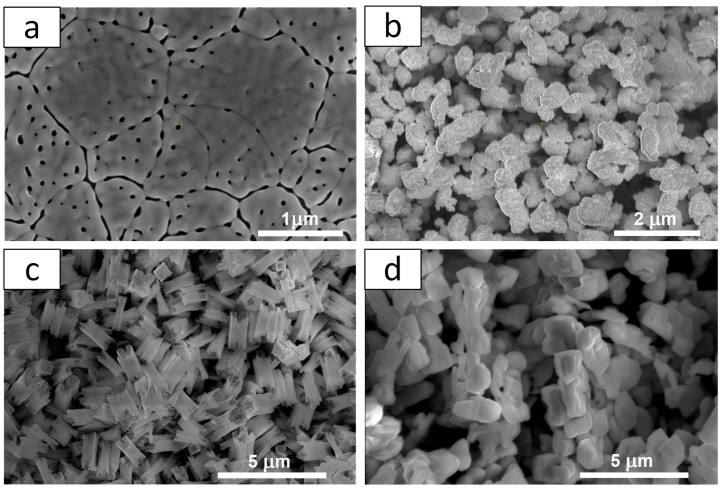
SEM images of BiVO_4_ prepared by different techniques: (**a**) l-BiVO_4_; (**b**) g-BiVO_4_; (**c**) s-BiVO_4_; and (**d**) m-BiVO_4_.

The resulting materials differ in structure and morphology. Figure 3 shows the SEM images of all materials. It is clearly visible that the l-BiVO_4_ material is obtained as a thin film that forms a compact, continuous layer with visible grain boundaries and some porosity distributed within and at the grain boundaries. The g-BiVO_4_ powder exhibits a homogeneous, aggregated morphology in the form of ovals with a relatively fine grain size (0.076 ± 0.019 µm). These primary grains tend to cluster into agglomerates with an average size of 0.46 ± 0.13 µm. The microstructure of s-BiVO_4_ powder reveals distinctive cuboid-like crystallites. The average cuboid dimensions are approximately 1.30 ± 0.07 µm (longer edge) and 0.53 ± 0.16 µm (shorter edge). The particles are relatively larger and appear to be more faceted and well defined, with a more uniform distribution. The powder particles of m-BiVO_4_ are larger and appear to be more faceted and well defined, with a more uniform distribution. The average particle size is about 0.59 ± 0.08 µm.

**Figure 4:**
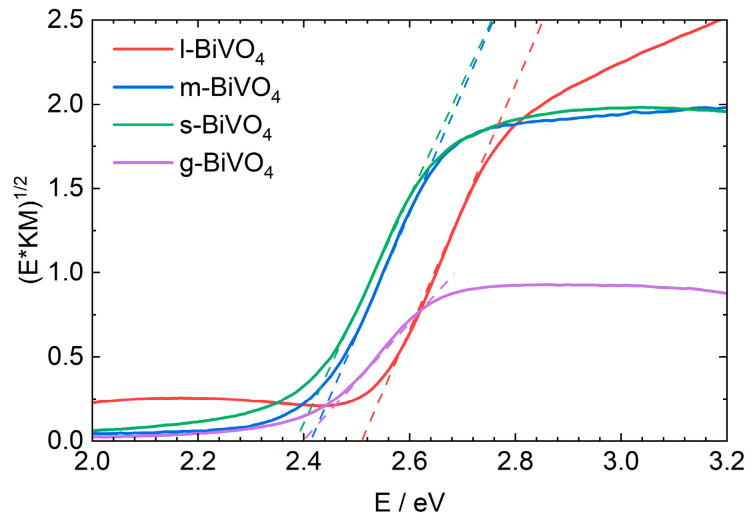
UV-Vis spectra of solid-state samples for different forms of BiVO_4_.

Based on the results obtained from XRD measurements, it is known that all samples crystallize in the monoclinic system, so their energy gap width is expected to be approximately 2.4 eV. To prove this, diffuse reflectance spectra were measured for all samples, and the energy of the band gap was determined using the Tauc method (Figure 4) [42]. The largest energy gap of 2.51 eV (494 nm) was observed for l-BiVO_4_, while the smallest of 2.38 eV was determined for s-BiVO_4_. g- and m-BiVO_4_ have almost identical energy gaps of 2.40 and 2.41 eV, respectively.

### 2.2. Photoelectrochemical Characterization

The next stage of this study was to verify the photocurrent generation efficiency of electrodes made from the obtained materials. Electrodes made from m-BiVO_4_, s-BiVO_4_, and g-BiVO_4_ were prepared by suspending the obtained powders in ethanol and then drop-casting them onto the surface of a foil coated with ITO, which served as a conductive substrate. The average thickness of the materials on the electrode measured by the profilometer were 3.82 ± 0.06 µm, 4.32 ± 0.07 µm, and 4.38 ± 0.05 µm for g-, s-, and m-BiVO_4_, respectively. In the case of l-BiVO_4_, the obtained material was a thin layer with a thickness of 115 ± 21 nm and required no further processing.

In the case of photoelectrodes, electrode porosity is an equally important issue, as it determines the size of the material’s active surface area. Therefore, Atomic Force Microscopy (AFM) images were obtained for all electrodes prepared from our materials.

**Figure 5:**
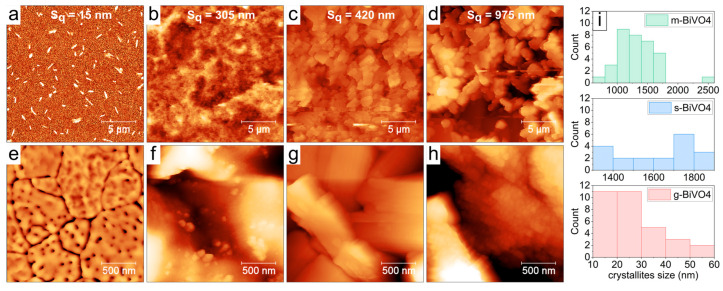
The AFM images of the surface of BiVO_4_ layers prepared by different synthesis methods: (**a**) l-BiVO_4_; (**b**) g-BiVO_4_; (**c**) s-BiVO_4_; and (**d**) m-BiVO_4_. All scans are 20 × 20 μm in size. The second row, (**e**–**h**), demonstrates small-scale details of all surfaces on 2 × 2 μm scans. (**i**) Distribution of crystallites sizes for g-BiVO_4_, s-BiVO_4_, and m-BiVO_4_ samples, evaluated from 20 × 20 μm scans.

Figure 5 presents the surfaces of BiVO_4_ layers obtained using different synthesis methods. The scans are arranged in an order of increasing surface roughness. The smoothest surface was observed for the l-BiVO_4_ sample with an RMS (Root Mean Square) roughness S_q_ of ~15 nm. It has a porous structure as shown in Figure 5e. These uncommon features visible in Figure 5a are narrow and steep, with heights in the range of 80–160 nm, and we attribute these steep features to some material agglomeration during the heating process after spin-coating. The g-BiVO_4_ sample of a medium roughness of S_q_~300 nm exhibits a “bubble shape” morphology with small spherical nano-crystallites (diameter: 27 ± 12 nm). The surface of s-BiVO_4_ has a roughness of S_q_~420 nm and consists of large rectangular-shaped crystallites (longer edge size: 1.63 ± 0.2 μm). And, finally, the surface of m-BiVO_4_ has the highest roughness of S_q_~975 nm and consists of smaller and more rounded crystallites with a size of 1.26 ± 0.25 μm. The large, micrometer-sized crystallites of both s-BiVO_4_ and m-BiVO_4_ samples are decorated with smaller round-shaped crystallites with an average size of 47 ± 13 nm and 77 ± 10 nm, respectively. The differences we report likely stem from variations in film formation dynamics associated with each preparation method. The spin-coating method promotes uniform spreading due to centrifugal force but can also lead to local instabilities or agglomeration during solvent evaporation, particularly during post-deposition thermal treatment. Hence, the layers obtained from l-BiVO_4_ are the thinnest but have numerous pores. In contrast, in the case of other materials obtained by drop-casting methods, slower solvent evaporation and different drying kinetics can influence the thickness of the film and cause greater grain aggregation on the ITO surface.

**Figure 6:**
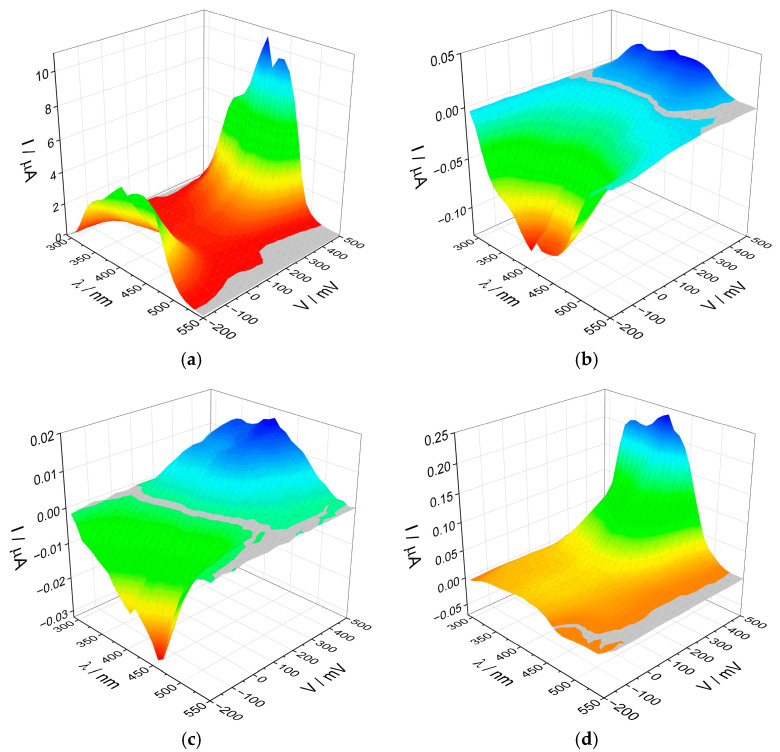
The photocurrent maps recorded for (**a**) l-BiVO_4_, (**b**) g-BiVO_4_, (**c**) s-BiVO_4_, and (**d**) m-BiVO_4_.

Chronoamperometric measurements were performed to determine the efficiency of photocurrent generation. All obtained electrodes were measured in a three-electrode system, where the working electrode was a layer of the synthesized materials coated on ITO, the reference electrode was a silver chloride electrode, and the auxiliary electrode was a platinum wire. A 0.1 mol/dm^3^ K_2_SO_4_ solution was used as an electrolyte. For all materials, the photocurrent response was measured in a wide potential range (from −200 to +500 mV, with every 50 mV). At each potential, the electrodes were pulse-illuminated in the range of 300–500 nm (in 10 nm steps) from the ITO side using a low-power halogen lamp (maximum 3 mW/cm^2^). Three-dimensional maps were created to illustrate the range of the response of our electrodes (Figure 6).

Surprisingly, despite minor differences in structure and band gap energy, the recorded photocurrent maps differ significantly for each material. The most noticeable differences are in the values of generated photocurrents. For the l-BiVO_4_ material, the maximum anodic photocurrent values exceed 10 µA, while for the other three materials, they are below 0.3 µA. This is likely because l-BiVO_4_ was obtained directly on a conductive substrate, ensuring good contact between the semiconductor grains and the substrate, facilitating charge transfer. It is well established that the efficiency of photocurrent generation is closely linked to the lifetime of excited-state carriers and their recombination dynamics. Electrodes fabricated from powder materials tend to be more porous, which promotes recombination processes and hinders effective charge transfer between grains, resulting in significantly lower photocurrent intensities.

The second difference between the materials is the appearance of the photoelectrochemical switching effect (PEPS effect) [43,44] in the g-BiVO_4_ and s-BiVO_4_ materials with a change in potential. BiVO_4_ is an *n*-type semiconductor, so we should observe only anodic photocurrents. However, in the case of these two materials, cathodic photocurrents (characteristic for *p*-type semiconductors) appear at potentials of +350 mV and +150 mV for g-BiVO_4_ and s-BiVO_4_, respectively. In the case of m-BiVO_4_, a small photocurrent switching was also observed, this time with a constant applied potential, but with a change in the wavelength of the incident light.

**Figure 7:**
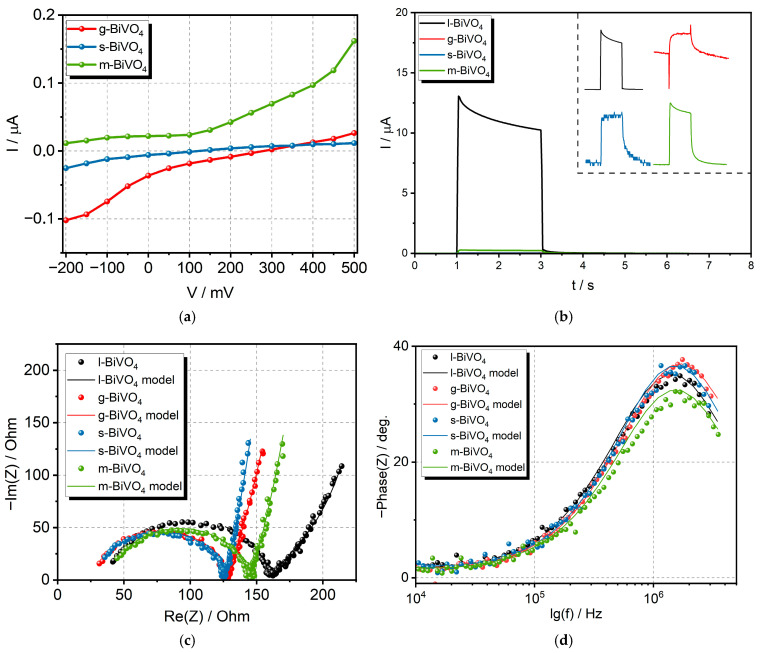
Results of (**a**) correlation between I and V under illumination with light (450 nm, 3 mW/cm^2^), (**b**) transient photocurrent for all samples measured under the applied potential of 0.5 V and the wavelength of 410 nm (inset shows the shape of pulse for each material), (**c**) Nyquist plot, and (**d**) Bode plots for all BiVO_4_ electrodes.

The exact photocurrent switching point, along with the potential change, can be determined by plotting the dependencies of the generated photocurrents on the applied potential at a single wavelength of light (450 nm). Figure 7a shows such dependencies for the g, s, and m-BiVO_4_ samples. We also analyzed the transient photocurrents for all samples during the illumination of the electrode. Under ideal conditions, the signal should rise rapidly to its maximum value when the light is turned on. Then, the current should remain constant during illumination and then decrease to zero when the light is turned off. However, the shape of this spectrum can be distorted due to electrode porosity, thickness, light intensity, or the presence of structural defects [45]. The obtained data measured under the applied potential of 0.5 V and the wavelength of 410 nm showed the difference in the profile of peaks for each material (see inset in Figure 7b). The profiles recorded for l- and m-BiVO_4_ are similar. We observe a rapid increase in the signal after switching on the light, followed by a slow decrease during illumination, which may be caused by surface charge effects generated by the accumulation of some impurities on the surfaces. The accumulated ions influence the interface band bending and increase the Schottky barrier height, which could be the reason for the observed photocurrent loss from the maximum value. After the light is turned off, the signal decreases and the accumulated surface charge is discharged. This process is slightly slower in the case of m-BiVO_4_. This type of photocurrent profile is called capacitive [46]. The situation is different for the photocurrent profiles recorded for g- and s-BiVO_4_, where, after turning on the light, we observe a slower increase in photocurrent to a maximum value throughout the illumination period, followed by a slow signal decline after turning off the light. This shape of the photocurrent peak is referred to as the inductive model [46]. In the case of g-BiVO_4_, a huge overshoot was observed, which could be interpreted as evidence of surface electron–hole recombination. It could explain the low intensity of generated photocurrents [47].

The dynamics of charge transfer was also analyzed using the electrochemical impedance spectroscopy (EIS) measurements. The EIS spectra obtained in current studies, visible in Figure 7c, were analyzed using an equivalent circuit described as (*R_s_* + *Q*/(*R_ct_* + *Z_B_*), where $R_{s}$—the contact series resistance; $R_{ct}$—the charge transfer resistance; *Q*—the constant phase element; and *Z_B_*—the Bisquert element. Standard capacitance was replaced with the constant phase element (CPE) to eliminate the influence of surface inhomogeneity (differences in chemical composition, stoichiometry, morphology, concentration of defects, and adsorption processes) [48]. The impedance of the CPE is expressed by Equation (1).

(1) $$Z_{CPE}=\frac{1}{Q{\left(j2\pi f\right)}^{\alpha }}$$

where *Q* is the CPE (F∙s^α−1^), *f* is the frequency in Hz, *j* is the imaginary unit (*j*^2^ = −1), and α is a constant (0 < *α* < 1, for a capacitor *α* = 1). In the obtained spectra, the phase angle associated with the low-frequency diffusive region substantially exceeds −*π*/4. Consequently, the Bisquert element (*Z_B_*), which describes anomalous linear diffusion, was utilized for fitting [48]. The mathematical form of this impedance element is detailed in the work [48] (see Equation (2)).

(2) $$Z_{B}=R_{B}\frac{{coth(\tau_{B}j2\pi f)}^{\gamma /2}}{{\left(\tau_{B}j2\pi f\right)}^{1-\gamma /2}}$$

where *γ* ≤ 1, $\tau_{B}$ is the diffusion time constant, $R_{B}$ is the resistance associated with the diffusion mechanism, *f* is the frequency in Hz, and *j* is the imaginary number *(j*^2^ = −1).

The resistance corresponding to charge transfer at the electrode–electrolyte interface varies from 92.7 Ω (s-BiVO_4_) to 117.9 Ω (l-BiVO_4_), indicating that the charge transfer resistance affecting the kinetics of the photogenerated charge carriers’ movement is strongly dependent on synthesis conditions (Figure 7c). The lowest value is for s-BiVO_4_, indicating that this material has the most effective electron transport. This is consistent with the transient photocurrent results, as the peak obtained for this material is most closely rectangular. The Bode plot presented in Figure 7d was utilized for the determination of the photoexcited electron lifetime (*τ_n_*). The electron lifetime was calculated based on the observed peak frequency (*f_max_*) corresponding to charge transfer at the material/electrolyte interface using Equation (3) [49]:

(3) $$\tau_{n}=\frac{1}{2\pi f_{max}}$$

The determined values were very close and amounted to 0.09–0.12 µs. All parameters corresponding to the utilized model, as well as determined electron lifetimes, were summarized in Table 1.

### 2.3. Mechanism of Photocurrent Generation

For a better understanding of the significant differences observed in photoelectrochemical properties, it is important to examine the electronic structure of the materials. The knowledge of the work function and Fermi level is crucial for all semiconductors. Therefore, for each material, contact potential difference (CPD) measurements were performed using a Kelvin probe in air at the same temperature (T = 22 °C) and humidity (34%). Based on the obtained results, the work function and the Fermi level for each material were calculated and are summarized in Table 2. (The detailed procedure for determining WF is described in Section 3.3). The obtained work function values range from 5.03 to 5.67 eV, which is generally consistent with the literature values for BiVO_4_, typically between 4.68 and 5.3 eV [50,51].

**Figure 8:**
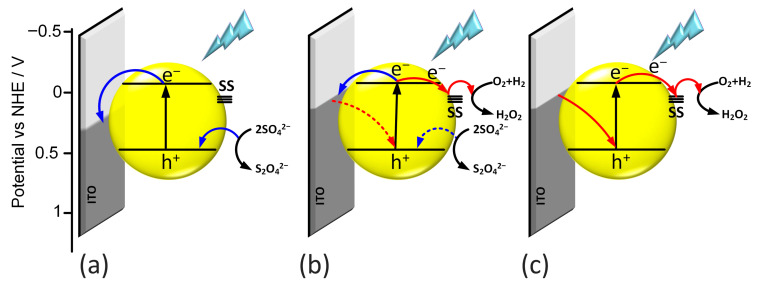
Mechanism of generation of anodic photocurrent (**a**), photocurrent switching (**b**), and cathodic photocurrent (**c**).

By analyzing all the obtained results, a mechanism for generating photocurrents was proposed for all materials. During the illumination of the photoelectrode, electron–hole pairs are generated at the surface of the electrode. Generally, electrons are transferred to the ITO substrate, while holes migrate toward the electrolyte interface. When there is good contact between the semiconductor and the conductive substrate, as observed in the case of the thin-layer l-BiVO_4_, the electron transfer occurs more efficiently, resulting in a higher photocurrent intensity. Simultaneously, the holes can react with SO_4_^2−^ anions present in the electrolyte (Figure 8a).

In contrast, for electrodes with a higher surface roughness, surface processes play a much more significant role in photocurrent generation. Additionally, light and applied potential can contribute to photocorrosion and lead to the formation of oxygen vacancies on the BiVO_4_ surface. These vacancies may introduce sub-band gap energy levels that act as trap centers, further influencing charge carrier dynamics [18]. In this situation, the pathway of photogenerated electron–hole pairs differs. Electrons from the conduction band can become trapped in sub-band gap states and subsequently react with species in the electrolyte. The presence of these additional trap states helps to explain the photocurrent switching effect observed in g-BiVO_4_ and s-BiVO_4_.

Photocurrent mapping reveals that the potential of photocurrent switching lies approximately 0.7 eV below the Fermi level. As a result, in the case of m-BiVO_4_, the photocurrent switching effect is only weakly pronounced. When the potential applied to the electrode is lower than the trap level, only anodic photocurrent is observed (Figure 8a). Conversely, if the applied potential is higher than the trap level, cathodic photocurrent is generated (Figure 8c). When the applied potential matches the energy level of the trap state, a wavelength-dependent switching of the photocurrent can be observed, as demonstrated for m-BiVO_4_ (Figure 8b).

## 3. Materials and Methods

### 3.1. Synthesis of Materials

l-BiVO_4_: A saturated solution of vanadyl acetylacetonate (Sigma-Aldrich, St. Louis, MO, USA) and bismuth nitrate (Bi(NO_3_)_3_, Aktyn, Bielsko-Biala, Poland) was prepared in a 1:1 mixture of DMF (N,N-dimethylformamide, Eurochem BGD, Tarnów, Poland) and DMSO (dimethyl sulfoxide, POCH S.A., Gliwice, Poland). The solution was made by adding 0.3 moles of substrates to 4 mL of the DMF-DMSO mixture, which was then stirred at 150 °C for two hours. The pH after mixing the substrates was 7.5, and its changes were not monitored during the synthesis. After stirring, the mixture was cooled, and the undissolved substrates were removed using a syringe filter. The layer was applied to the clean ITO (indium tin oxide, Ossila, Sheffield, UK) glass using the spin-coating method and was placed on a hot plate at 100 °C for preliminary drying. Once the preliminary drying was complete, the layer changed color from green to yellow. In the final step, the layer was placed in a furnace for 2 h at 450 °C. The furnace was heated at a rate of 2 °C per minute until reaching 450 °C, after which the sample was maintained at this temperature for 2 h, and then cooled without any temperature program.

g-BiVO_4_: A 0.1 mole quantity of sodium orthovanadate (NaVO_4_, BDH) was dissolved in 48 mL of distilled water with the addition of 2 mL of 25% ammonia (NH_3_, POCH S.A.). Next, 0.1 moles of bismuth nitrate were dissolved in 46 mL of distilled water with 12 mL of 2 mol/dm^3^ nitric acid (HNO_3_, Chempur, Mumbai, India). After mixing both solutions, 20 mL of 0.1 mol/dm^3^ citric acid (POCH S.A.) was added. After 10 min, the mixture was neutralized using ammonia. The next step involved stirring for 5 h at 90 °C to evaporate the water and form a gel. The final step was calcination at a heating rate of 2 °C per minute until reaching 250 °C and calcination at this temperature for 3 h, then cooling without any temperature program.

m-BiVO_4_: A 0.001 mole quantity of sodium orthovanadate (NaVO_4_, BDH) was dissolved in 25 mL of distilled water with the addition of 4 mL of a 2 mol/dm^3^ sodium hydroxide solution (NaOH, POCH S.A.). Bismuth nitrate (Bi(NO_3_)_3_, Aktyn) was dissolved in 25 mL of distilled water with the addition of 5 mL of 2 mol/dm^3^ nitric acid (HNO_3_, Chempur). Both solutions were mixed and placed in a Teflon vessel in a microwave reactor (Magnum II, Ertec, Wrocław, Poland) with controlled temperature and pressure for 90 min at a temperature of 255 °C and a pressure of 44 bar. The maximum power of the device was 600 W and was automatically changed during the synthesis to maintain a constant temperature and pressure value. After the synthesis was completed, the mixture was gradually cooled over the next 90 min until it reached 25 °C and atmospheric pressure. After the microwave process, the final product was centrifuged, washed several times with distilled water, and dried at 70 °C.

s-BiVO_4_: A 0.001 mole quantity of sodium orthovanadate (NaVO_4_, BDH) was dissolved in 25 mL of distilled water with the addition of 4 mL of a 2 mol/dm^3^ sodium hydroxide solution (NaOH, POCH S.A.). Bismuth nitrate (Bi(NO_3_)_3_, Aktyn) was dissolved in 25 mL of distilled water with the addition of 5 mL of 2 mol/dm^3^ nitric acid (HNO_3_, POCH S.A). Both solutions were mixed, forming an orange suspension. The mixture was then neutralized (pH = 7) using 2 mol/dm^3^ NaOH. Next, the solution was placed in a Bandelin sonochemical reactor (Bandelin Electronic GmbH & Co. KG, Berlin, Germany). The reaction was initiated using a VS70T sonotrode (Bandelin Electronic GmbH & Co. KG, Berlin, Germany) at 70% (0.3 kJ) power and continued for 90 min. At the end, the product was centrifuged, washed several times with distilled water, and dried at 70 °C for 12 h.

### 3.2. Preparation of Working Electrode

Electrodes for photoelectrochemical experiments were prepared according to the following procedure. First, 10 μg of bismuth vanadate was added to 1 mL of ethanol. The mixture was then sonicated in an ultrasonic cleaner for 30 min. After that, an ITO foil (indium tin oxide, Sigma Aldrich) was placed on a hot plate at 100 °C, and the ethanol–bismuth vanadate mixture was drop-cast three times, with each drop containing 35 μL of the mixture.

### 3.3. Characterization Techniques

Diffuse reflectance spectroscopy (DRS): The spectra for solid-state samples were measured on a LAMBDA 750 UV/vis/NIR spectrophotometer equipped with a 100 mm integrating sphere (PerkinElmer Inc., Springfield, IL, USA). Spectra were recorded for powder samples prepared by grinding the mixture of powder samples with BaSO_4_ in an agate mortar. BaSO_4_ was used as a reference sample.

X-ray diffraction (XRD): Bragg–Brentano X-ray diffraction measurements were carried out using a PANalytical Empyrean diffractometer with Cu Kα radiation (Kα_1_ = 1.541 Å, Kα_2_ = 1.544 Å) in the reflection mode. Parallel beam geometry was employed to enhance measurement accuracy. The incident beam optics included a Goebel mirror, a Soller slit (2.29°), and a fixed divergence slit (1/2°). The diffracted beam optics were equipped with a parallel plate collimator (0.18°) and an additional Soller slit (2.29°).

Scanning Electron Microscopy (SEM): The surface morphology and microstructure of the BiVO_4_ samples were examined without conductive coating, using low-voltage conditions (1 kV) in a Thermo Scientific Helios 5 scanning electron microscope (SEM) (Waltham, MA, USA) or a low-vacuum mode (70 Pa water vapor pressure, 10–15 kV) in an FEI Versa 3D SEM. The SEM imaging was performed in a secondary electron (SE) mode to visualize the surface topography. Depending on the instrument and imaging conditions, the Through-Lens Detector (TLD), Low-Vacuum Secondary Electron Detector (LVSED), and Everhart–Thornley Detector (ETD) were employed to optimize the contrast and resolution of the layers and powder particles. The average particle size was determined from the SEM images using the ImageJ 1.53 software. To ensure statistical relevance, at least 50 individual particles were measured for each sample.

Atomic Force Microscopy (AFM): The topographic images of samples were obtained using the Atomic Force Microscope (Bruker Dimension ICON XR, Cambridge, MA, USA) in the PeakForce QNM (quantitative nanomechanical mapping) mode with the SCANASYST-AIR probe (nominal tip radius: 2 nm) at ambient air conditions. The size of the scans varied from 20 × 20 µm to 500 × 500 nm. For AFM scan processing, we employed the Gwyddion 2.67 software tool. A total of 19 to 34 clearly distinguishable particles were manually analyzed per sample, depending on scan quality and particle distinguishability. For non-spherical particles, the longer axis was taken as the reference dimension.

Work function measurements (WF): The Kelvin probe (KP) measurements were performed with the use of a commercially available probe (KP Technology, Caithness, Scotland), which contains a stainless steel tip (with a diameter of 5 mm) attached to a cantilever (connected to an electromagnetic transducer), which oscillates with an amplitude of 70 mm at a frequency of 72 Hz in ambient air. In this configuration, the Contact Potential Difference (CPD) between sample and tip is measured with a resolution better than 3 meV, and then recalculated for the absolute work function value. To determine the value of the work function for the tip, the CPD for the gold reference sample (WF = 5.1 eV) was measured and calculated according to the equation: WF(tip) = WF(gold) − CPD. To determine the work function for our samples, we measured CPD and calculated it according to the following equation: WF(sample) = WF(tip) + CPD. Measurements for all samples were carried out under the same conditions: T = 22 °C, humidity = 34%

Profilometry: The thickness of thin layers deposited on ITO was measured by a contact needle profilometer (Bruker DektakXT profilometer, Cambridge, MA, USA).

### 3.4. Photoelectrochemical Measurements

Photocurrent measurements: Photoelectrochemical (chronoamperometry with on/off light pulses, CA) measurements were performed in a standard three-electrode system consisting of the leakless Ag/AgCl reference electrode, the Pt wire counter electrode, and the obtained samples as the working electrode. The 0.1 mol/dm^3^ solution of potassium sulfate (K_2_SO_4_) was used as an electrolyte. Measurements were performed using a custom-built photocurrent spectrometer with a xenon lamp (Instytut Fotonowy, Krakow, Poland).

Electrochemical Impedance Spectroscopy: EIS measurements were performed using a Biologic SP-300 potentiostat/galvanostat (Bio-Logic Science Instruments, Seyssinet-Pariset, France) equipped with the EIS module and M470 work station. The measurements were carried out using the standard three-electrode setup (a Pt gauze as the counter electrode and an Ag/AgCl leakless electrode as the reference electrode). The synthesized materials were deposited onto the surface of the ITO glass substrates and then applied as working electrodes. The electrochemically active surface area of the electrodes amounted to 0.5 cm^2^. The EIS spectra were recorded at −0.1 V (vs. Ag/AgCl) in a 0.1 mol/dm^3^ Na_2_SO_4_ solution purged for 15 min with Ar. Measurements were performed at frequencies ranging from 7 MHz to 0.1 kHz and a potential amplitude of 10 mV under dark conditions.

## 4. Conclusions

The obtained results indicated that the final physicochemical properties are strongly dependent on the synthesis route. The SEM observations reveal the influence of the synthesis route on BiVO_4_ particle morphology, ranging from fine-grained aggregates (g-BiVO_4_) to cuboid-like structures (s-BiVO_4_) and larger, faceted particles with uniform sizes (m-BiVO_4_). Our results suggest that subtle changes in particle size, shape, and surface structure can influence the optical properties of BiVO_4_, even when the crystallographic phase is identical. The UV-Vis spectra revealed that, independent of the applied synthesis method, the optical band gap determined for all obtained materials is very close. Despite similar optical band gaps, materials exhibit significantly different work function values, indicating the differences in the Fermi level localization. The specific band structure of the obtained materials was indicated as the main factor affecting the mechanisms and efficiency of photocurrent generation.

The highest photocurrent generation efficiency was achieved with thin-layer BiVO_4_ synthesized from an organic precursor directly on the ITO substrate, ensuring excellent contact with the surface. These films are transparent, smooth, durable, and well suited for use as electrodes in photoelectrochemical measurements. In contrast, the other materials—g-BiVO_4_, s-BiVO_4_, and m-BiVO_4_—produced lower-intensity photocurrents; however, the observed photocurrent switching effect indicates their potential application in molecular logic gates or memory devices. Our results show that selecting the synthesis method may be considered as a promising way of designing BiVO_4_-based materials for devices based on the photoactive materials of specific band structure and thus photoelectrochemical characteristics.

## Figures and Tables

**Table 1:** Summary of parameters determined based on recorded EIS spectra.

Sample	*R_s_* Ω	*Q* F∙s^α−1^	α	*R_ct_* Ω	*R_B_* Ω	*τ_B_* s	*γ_B_*	*f_max_* MHz	*τ_n_* µs
l-BiVO_4_	40.9	2.58 × 10^−9^	0.98	117.9	303.8	1.607 × 10^−2^	0.74	1.36	0.12
g-BiVO_4_	30.4	2.52 × 10^−9^	0.99	95.7	481.1	9.224 × 10^−3^	0.41	1.72	0.09
s-BiVO_4_	31.3	2.23 × 10^−9^	1	92.7	202.6	1.919 × 10^−3^	0.38	1.38	0.12
m-BiVO_4_	41.5	2.60 × 10^−9^	0.98	101.8	350.4	5.449 × 10^−3^	0.40	1.78	0.09

**Table 2:** The band gap, work function (WF) values, and potential of the Fermi level (E_F_) of the studied BiVO_4_ samples.

Sample	Band Gap eV	WF eV	E_F_ V
l-BiVO_4_	2.51	5.35	0.85
g-BiVO_4_	2.40	5.67	1.17
s-BiVO_4_	2.38	5.23	0.73
m-BiVO_4_	2.41	5.03	0.53

## Data Availability

The original contributions presented in this study are included in the article. Further inquiries can be directed to the corresponding authors.
